# Methylglyoxal Induced Basophilic Spindle Cells with Podoplanin at the Surface of Peritoneum in Rat Peritoneal Dialysis Model

**DOI:** 10.1155/2015/289751

**Published:** 2015-05-03

**Authors:** Ichiro Hirahara, Hideki Sato, Toshimi Imai, Akira Onishi, Yoshiyuki Morishita, Shigeaki Muto, Eiji Kusano, Daisuke Nagata

**Affiliations:** ^1^Department of Nephrology, Jichi Medical University, 3311-1 Yakushiji, Shimotsuke, Tochigi 329-0498, Japan; ^2^Terumo Core Technology Cente, 1500 Inokuchi, Nakai-machi, Ashigarakami-gun, Kanagawa 259-0151, Japan

## Abstract

Peritoneal dialysis (PD) is a common treatment for patients with reduced or absent renal function. Long-term PD leads to peritoneal injury with structural changes and functional decline. At worst, peritoneal injury leads to encapsulating peritoneal sclerosis (EPS), which is a serious complication of PD. In order to carry out PD safely, it is important to define the mechanism of progression of peritoneal injury and EPS. We prepared rat models of peritoneal injury by intraperitoneal administration of glucose degradation products, such as methylglyoxal (MGO) or formaldehyde (FA), chlorhexidine gluconate (CG), and talc. In rats treated with MGO, peritoneal fibrous thickening with the appearance of basophilic spindle cells with podoplanin, cytokeratin, and *α*-smooth muscle actin at the surface of the peritoneum was observed. These cells may have been derived from mesothelial cells by epithelial-to-mesenchymal transition. In FA- or CG-treated rats, the peritoneum was thickened, and mesothelial cells were absent at the surface of the peritoneum. The CG- or MGO-treated rats presented with a so-called abdominal cocoon. In the talc-treated rats, extensive peritoneal adhesion and peritoneal thickening were observed. MGO-induced peritoneal injury model may reflect human histopathology and be suitable to analyze the mechanism of progression of peritoneal injury and EPS.

## 1. Introduction

Long-term peritoneal dialysis (PD) leads to peritoneal injury with functional decline, such as ultrafiltration loss. Peritoneal injury is often accompanied by histological changes, such as peritoneal fibrosis and sclerosis. At an early stage of peritoneal injury, epithelial-to-mesenchymal transition (EMT) of mesothelial cells is induced at the surface of the peritoneum, followed by evident diffuse fibrous thickening, neovascularization, and mononuclear cell infiltration [1, 2]. At worst, peritoneal injury leads to encapsulating peritoneal sclerosis (EPS), a serious complication of PD [3–7]. At advanced stages of EPS, the small intestine adheres and is encapsulated within a thick collagen-rich peritoneum to form a cocoon-like mass. EPS is associated with not only deterioration of peritoneal function but also clinical symptoms, such as ileus. EPS occurs in about 0.4%–3.3% of patients who undergo PD. However, EPS has a high mortality rate, and about half of the patients with EPS die [4–7]. The causes of peritoneal injury and EPS have not been clarified, but they appear to develop through the interaction of multiple factors, such as infection with bacteria or fungi resulting in peritonitis; antiseptics; exogenous materials like particulates and plasticizers; and continuous exposure to nonphysiological PD solutions having high concentrations of glucose and glucose degradation products (GDPs), low pH, and high osmolarity [4, 7]. These factors may induce fibrosis, sclerosis, inflammation, angiogenesis, and vasculopathy in the peritoneum. The administration of corticosteroids, tamoxifen, and immunosuppressive agents and total parenteral nutrition are effective in an early stage of EPS development [4–6]. However, for an advanced stage of EPS, in which bowel adhesions have formed, the only effective therapeutic method is surgical dissection of the encapsulated peritoneum; this must be performed by skilled surgeons using specialized techniques [4–6]. Therefore, there is a compelling need for methods for the early diagnosis of EPS. Animal models are often used to define the mechanism of progression of peritoneal injury and the evaluation of drugs or screening of predictive markers for EPS.

The aim of this study is to advance a peritoneal injury animal model reflecting human histopathology in order to elucidate the mechanism of progression of peritoneal injury. To perform PD safely, it is important to define the mechanism of pathogenesis and progression of peritoneal injury and EPS, and then it is necessary to prevent peritoneal injury from developing into EPS.

## 2. Materials and Methods

### 2.1. Preparation of Animal Models of Peritoneal Injury

We prepared four animal models that reflected the pathology of peritoneal injury. All of the groups were treated with intraperitoneal injections of various solutions. Animals used in this study were male Sprague-Dawley rats (5 to 6 weeks of age, weighing about 200 to 250 g; Charles River Japan, Kanagawa, Japan; *n* = 6/group).

Rats in the groups treated with GDPs, such as methylglyoxal (MGO) or formaldehyde (FA), intraperitoneally received 100 mL/kg/day GDP containing PD fluids (PDFs) for 21 days. The PDFs used in the present study were prepared by adding 20 mM MGO or FA to PDF (2.5% glucose, 100 mM NaCl, 35 mM sodium lactate, 2 mM CaCl_2_, and 0.7 mM MgCl_2_) and were then sterilized by filtration. The PDFs were prepared and adjusted to pH 5.0 just before injection every day. Rats in the chlorhexidine gluconate- (CG-) treated group intraperitoneally received 15 mL/kg/day 0.1% CG/15% ethanol/saline for 21 days. The solution was aseptically prepared. Rats in the talc-treated group intraperitoneally received 75 mL/kg/day talc suspension, which was prepared by dispersing 1 g of talc in 15 mL of saline followed by autoclave sterilization. The talc-treated group received one administration every 7 days for three weeks. The concentrations of MGO, FA, CG, and talc were decided based on previous reports [7–16]. As a control, a group treated with the PDF without adding MGO, FA, CG, or talc was also set. The control rats were given an intraperitoneal injection of 100 mL/kg/day PDF for 21 days. If solution remained in the peritoneal cavity, it was drained before the injection. When PDF had been injected for more than 3 weeks, it was difficult to inject it because of extensive peritoneal adhesion. Therefore, on the 22nd day after the start of the experiment, peritoneal equilibration test (PET) was performed. Subsequently, the parietal peritoneum was sampled for histological analysis from corresponding sites in each rat.

We performed our experiments in accordance with the NIH Guide for the Care and Use of Laboratory Animals. The animals were housed in an air-conditioned room at a constant temperature of 23 ± 2°C and a relative humidity of 50 ± 10% and kept under a 12-hour light/dark cycle with free access to sufficient pellet food and water. Adequate attention was paid to maintaining a hygienic environment and to preventing infectious peritonitis. Furthermore, a sterility test was performed using the dialysate drained for PET to check for the presence of aerobic bacteria, anaerobic bacteria, and fungi in drained dialysate, and then all rats were confirmed to be uninfected.

### 2.2. Peritoneal Equilibration Test (PET)

In order to analyze peritoneal function, the peritoneal permeability of glucose was estimated by PET. First, intra-abdominal fluid was drained out. After 50 mL/kg PDF containing 2.5% glucose had been intraperitoneally injected, drained dialysate was collected immediately at 0 minutes and at 90 minutes. The injection volume was set based on that used in a human clinical context. Glucose levels were determined by SRL Co., Ltd. (Tokyo, Japan). The ratio of the glucose level in drained dialysate obtained 90 minutes after the injection to that obtained immediately after the injection was defined as the *D*/*D*_0_ glucose level.

### 2.3. Histological Analysis

Left center parietal peritoneum was sampled from corresponding sites of each rat and was fixed with 10% FA/0.1 M phosphate buffer (pH 7.2). Peritoneal specimens were embedded in paraffin to prepare tissue sections of a thickness of 2-3 *μ*m. To determine the thickness of the peritoneum, the sections were sliced perpendicularly to the peritoneal surface. Each section was stained with hematoxylin-eosin (HE) to analyze cell type and with Azan to identify collagen fibers. The morphologic changes of the peritoneum were evaluated in a blind manner with photomicroscopy by a toxicological pathologist.

The thickness of the peritoneum was measured with image-analysis software (Win ROOF, Mitani Co., Fukui, Japan). The specimens of the peritoneum were obtained from two sites in each rat. The thickness was measured at 30 points per site (at 0.5 mm intervals within a range of 1.5 cm), and the average was calculated.

### 2.4. Immunohistochemistry

Peritoneal tissue samples embedded in paraffin were sectioned at a thickness of 2-3 *μ*m. These sections for analysis of cytokeratin or *α*-smooth muscle actin (*α*-SMA) were dewaxed with xylene. After endogenous peroxidase activity had been blocked with 3% hydrogen peroxide/methanol for 15 minutes, the sections were treated with 0.25% trypsin/1 mM EDTA for 2 hours at room temperature. These sections were treated for 2 hours at room temperature with a monoclonal antibody against *α*-SMA (Sigma Chemical Co., St. Louis, MO, USA) at a dilution of 1 : 2000 to identify mesenchymal cells or for 2 hours at room temperature with prediluted monoclonal antibodies against pan-cytokeratin (Sigma Chemical Co.) at a dilution of 1 : 2 to identify mesothelial cells. For the analysis of podoplanin location, after dewaxing in xylene, the sections were autoclaved at 121°C for 10 min. After blocking with 3% hydrogen peroxide, the sections were treated overnight at 4°C with a rabbit polyclonal antibody against podoplanin (Bios Inc., Boston, MA, USA) at a dilution of 1 : 1000 to identify activated mesothelial cells. Sections were incubated with biotinylated anti-rabbit immunoglobulins for 30 min and then treated with streptavidin-horseradish peroxidase conjugate for 30 min followed by detection with DAB. These sections were also counterstained with Meyer's hematoxylin.

### 2.5. Statistical Analysis

Data are given as mean ± SD. Statistical analysis was performed by Dunnett's multiple comparison test. A *P* value of less than 0.05 was accepted as significant.

## 3. Results

**Figure 1:**
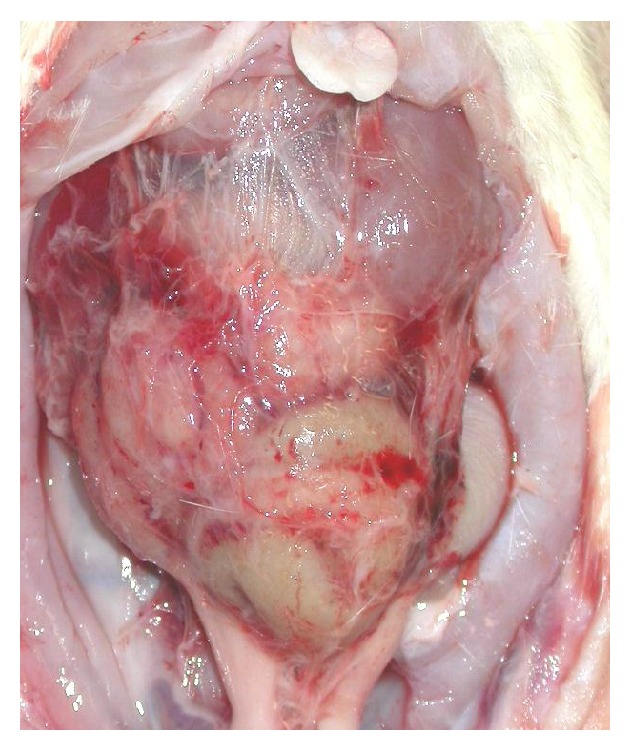
Appearance of organs in the peritoneal cavity in MGO-treated rat. The peritoneal injury models were prepared by repeated administration of MGO containing peritoneal dialysis fluids into the peritoneal cavity of rats for 21 days. Adhesion of bowel forming a cocoon-like mass was seen in the MGO-treated rats.

**Figure 2:**
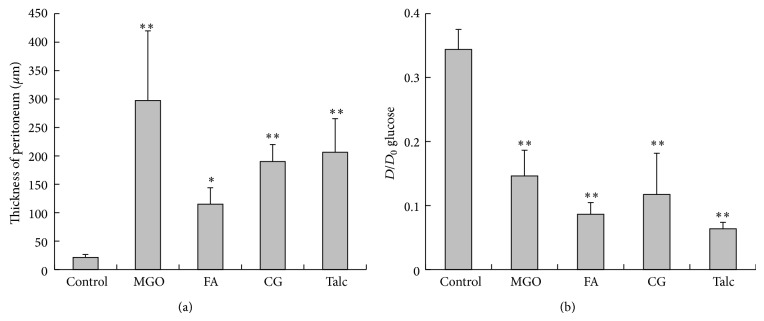
Peritoneal deterioration in the rat peritoneal injury models. (a) Thickness of parietal peritoneum. (b) The PET was performed in peritoneal injury animal models and *D*/*D*_0_ glucose level was obtained. Data are shown as mean ± SD. Statistical analysis was performed by Dunnett's multiple comparison test against the control. ^∗^ *P* < 0.05 compared with the control. ^∗∗^ *P* < 0.01 compared with the control.

**Figure 3:**
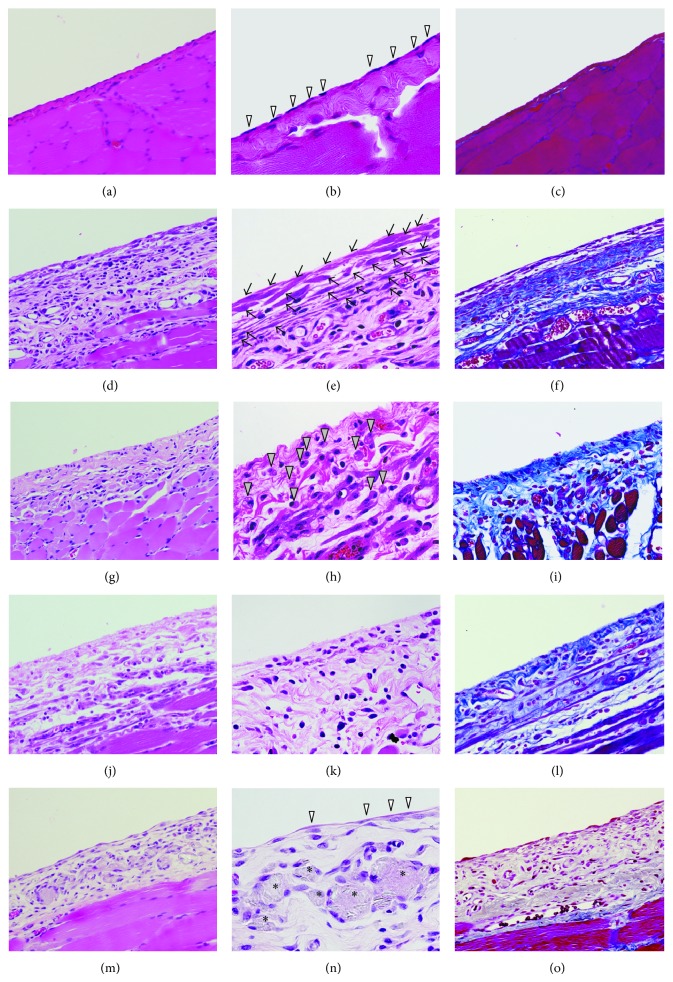
Histopathological findings of parietal peritoneum. The parietal peritoneum was analyzed histologically with HE stain (a, b, d, e, g, h, j, k, m, and n) or Azan stain (c, f, i, l, and o). Control rat: (a, b, and c). MGO-treated rat: (d, e, and f). FA-treated rat: (g, h, and i). CG-treated rat: (j, k, and l). Talc-treated rat: (m, n, and o). Mesothelial cells, spindle cells, phagocytosis by macrophages, and multinucleated giant cells are indicated by open arrow heads, arrows, closed arrow heads, and asterisks, respectively. (a, c, d, f, g, i, j, l, m, and o): ×200, (b, e, h, k, and n): ×400.

**Figure 4:**
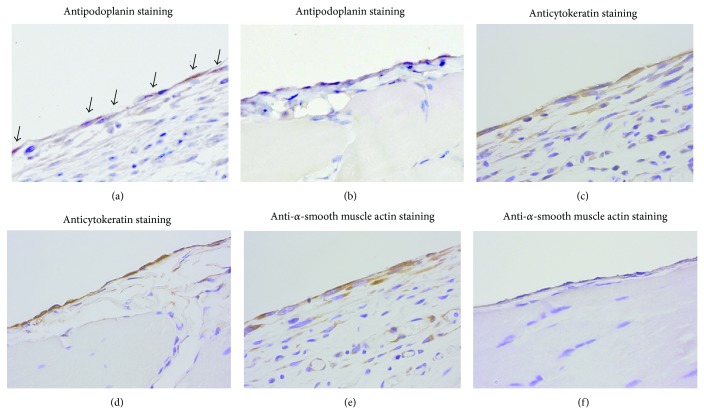
Characterization of cells at the surface of the peritoneum in the MGO-treated rats. In the parietal peritoneum, podoplanin-positive cells (a, b), cytokeratin-positive cells (c, d), and *α*-smooth muscle actin-positive cells (e, f) were analyzed by immunostaining. Podoplanin-positive cells are indicated by arrows (a). MGO-treated rat: (a, c, and e). Control rat: (b, d, and f). Magnification: ×400.

In the MGO-, FA-, and CG-treated rats, fist-like round liver edge and extensive adhesion of the bowel and stomach were observed. In the MGO- or CG-treated rats, the bowel adhesion, called an abdominal cocoon, was observed as a mass surrounded by thickened peritoneum (Figure 1). In the talc-treated rats, extensive adhesion of the peritoneum was observed, although the cocoon-like adhesion was not formed. By analysis of tissue sections of parietal peritoneum, peritoneal thickening occurred in the MGO-, FA-, CG-, and talc-treated rats. The thickness of the peritoneum in each group is shown in Figure 2(a). In the MGO-, FA-, CG-, and talc-treated groups, mononuclear cell infiltration, neovascularization, and fibrosis were observed in the peritoneum. In the MGO-treated rats, the peritoneal tissue consisted of dense collagen fibers. However, in the surface of the peritoneum, collagen was scarce, and basophilic spindle cells with podoplanin, cytokeratin, and *α*-smooth muscle actin proliferated excessively (Figure 4). Deposition of fibrin was observed in parts of the peritoneal surface. In the FA-treated rats, mesothelial cells were deciduated and then cells were absent at the surface of the peritoneum. In the CG-treated rats, phagocytosis by macrophages, edema, and loss of mesothelial cells were also observed. The surface of the peritoneum was covered with a fibrin layer in some rats. In the talc-treated rats, foreign body inflammation was confirmed by multinucleated giant cell formation from macrophage by phagocytosis of talc particle. In control rats, there was a single layer of mesothelial cells at the surface of the thin peritoneum. The morphology of the peritoneum is shown in Figure 3 and pathological findings of the peritoneum in peritoneal injury rats are summarized in Table 1. There was no difference in morphology between saline- and PDF-treated rats (data not shown).

Peritoneal permeability was higher in the MGO-, FA-, CG-, and talc-treated rats than in the control rats (Figure 2(b)).

## 4. Discussion

In the peritoneum of EPS patients, the frequencies of fibrin deposition, fibroblast swelling, capillary angiogenesis, and mononuclear cell infiltration were significantly higher than those of non-EPS patients [17]. There are many candidate factors that could cause peritoneal injury and EPS in PD, but the mechanism of peritoneal deterioration has not been clarified [3–7]. In the present study, we prepared four animal models for a comparison of the pathology of peritoneal injury induced by different factors, namely, MGO, FA, CG, and talc [7–16]. In these animal models, enhanced peritoneal permeability (Figure 2(b)) was confirmed and extensive peritoneal adhesion was found, as in human EPS patients [1–5] (Figure 1). Thickening of the peritoneum, neovascularization, and mononuclear cell infiltration were observed in the peritoneum of these animal models (Figure 2(a)). These findings are similar to the pathological picture of the peritoneum in human EPS [3, 18]. However, these models do not necessarily reflect the human clinical pathology. It is important to recognize the characteristics of each model.

The first and second peritoneal injury animal models were made by intraperitoneally administering the PDFs supplemented with MGO or FA, respectively [7–10]. Conventional PDFs contain various GDPs, such as 5-hydroxymethylfurfural, furaldehyde, acetaldehyde, FA, glyoxal, MGO, 3-deoxyglucosone, and 3,4-dideoxyglucosone-3-ene [11]. These GDPs contribute greatly to the bioincompatibility of conventional PDFs and are risk factors for EPS. Glucose is safe and readily metabolized because it is the most important basic source of energy for metabolism. Therefore, glucose is widely used as an osmotic agent in commercial PDFs. However, glucose in PDFs is degraded to GDPs during heat sterilization and storage [12]. The resulting GDPs show strong oxidant activity and toxic effects on cell proliferation and cell function [10]. In particular, carbonyl compounds, such as MGO, FA, and 3,4-dideoxyglucosone-3-ene, are extremely cytotoxic [7, 10, 11]. These GDPs form advanced glycation end products (AGEs) that induce structural changes of the peritoneum or the loss of peritoneal function [8]. GDPs injure the peritoneum directly or via the accumulation of AGEs. The concentrations of MGO and FA, injected into the MGO and FA models, are reasonable given the results of permitted daily exposure (PDE) risk assessment [7]. GDP-induced peritoneal injury models may reflect human PD. In particular, in the MGO-induced model, basophilic spindle cells are observed in the surface of the peritoneum at an early stage and a cocoon-like bowel adhesion is also formed at a late stage [7, 8]. Basophilic spindle cells may be derived from mesothelial cells via EMT-like change induced by carbonyl stress of MGO or accumulation of AGE. These morphological changes of human peritoneum were often reported in a clinical context. The MGO-induced model may be suitable to analyze the mechanism of progression of peritoneal injury and EPS.

The third animal model was made by intraperitoneally administering an antiseptic, CG, as a chemical irritant to induce inflammation. Phagocytosis by macrophages induced by aggregated grains of CG may be a trigger for this inflammation. This model is most often used to analyze peritoneal injury or EPS in animal studies [8, 9, 12–16]. However, it is thought that CG is not the main cause of peritoneal injury with thickening in PD patients because it is now scarcely used clinically as an antiseptic in this condition [9]. In addition, in this model, an extremely excessive dose of CG is administered compared with daily exposure in humans. Inflammation induced by CG caused the development of edema followed by fibrosis without EMT-like cells [8, 9, 12–16]. Therefore, the CG model is probably inadequate to analyze the mechanism of progression of peritoneal injury.

The fourth model was made by administering talc (hydrous magnesium silicate) as an exogenous material into the peritoneal cavity, since this agent has long been known to cause adhesion formation [14, 19]. In this peritoneal injury model, the volume of talc injected is extremely high and large particles such as talc are not observed in the human peritoneum. This model may thus not reflect the progression of human clinical peritoneal injury in PD.

In EMT, fully differentiated mesothelial cells undergo transition to a mesenchymal phenotype, a spindle-shaped morphology, concomitant with the acquisition of a mesenchymal marker, *α*-SMA. Some studies showed that the EMT of mesothelial cells may be a triggering factor of peritoneal injury in PD patients [1, 2]. Transforming growth factor-*β* (TGF-*β*) induces the EMT-like change of mesothelial cells via Snail, a zinc-finger transcription factor, and causes peritoneal fibrosis [1, 2, 8]. Podoplanin, which is a member of a type-1 transmembrane sialomucin-like glycoprotein family, serves as a marker of lymphatic endothelial cells but is also expressed by mesothelioma [19]. In the peritoneum of patients with EPS, podoplanin is expressed by activated mesothelial cells, lymphatic endothelial cells, and *α*-SMA-positive myofibroblasts. Braun et al. reported the possibility that podoplanin-positive myofibroblasts are derived from mesothelial cells by EMT and these cells are a hallmark of EPS [20, 21]. In the present study, basophilic spindle-shaped cells with podoplanin, cytokeratin, and *α*-SMA proliferated excessively in the MGO-treated rats. Podoplanin, cytokeratin, and *α*-SMA are markers for activated mesothelium, mesothelial cells, and mesenchymal cells, respectively. The basophilic spindle-shaped cells may be derived from mesothelial cells by EMT-like change. On the other hand, Hou et al. reported that podoplanin was expressed on a subset of F4/80(+) macrophages, a subset which they have termed fibroblastic macrophages [22]. Miyamoto et al. reported that podoplanin was an inflammatory protein upregulated in Th17 cells [23]. In the present study, the podoplanin-positive basophilic spindle-shaped cells at the surface of peritoneum may be derived from fibroblastic macrophages or Th17 cells. Additional studies are needed to clarify the origin of the spindle-shaped cells.

There is little evidence about podoplanin-positive spindle-shaped cells, even though podoplanin might be a suitable morphological marker supporting the diagnosis and might be involved in the pathogenesis of EPS. To our knowledge, the present study is the first to report that podoplanin-positive spindle-shaped cells are induced in PD animal models. The MGO-induced peritoneal injury model may provide valid information about the mechanism of pathogenesis and progression of peritoneal injury developing into EPS.

## 5. Conclusion

MGO induced the podoplanin-positive basophilic spindle cells at the surface of the peritoneum in a peritoneal injury animal model. This animal model may reflect the progression of peritoneal injury of human PD patients, so additional studies using this animal model may contribute to clarifying the mechanism of peritoneal deterioration.

## Figures and Tables

**Table 1:** Pathological findings of the peritoneum in peritoneal injury rats.

	MGO	FA	CG	Talc
Loss of monolayer of mesothelial cells	+++	+++	+++	−
Appearance of basophilic spindle cells	++	−	−	−
Phagocytosis in macrophages	−	−	+−	++
Foreign body inflammation	−	−	−	++
Edema	−	−	+	−
Mononuclear cell infiltration	++	+	++	++
Neovascularization	++	+	+	+
Fibrosis	+	+	+	+

MGO: methylglyoxal-treated rats, FA: formaldehyde-treated rats, CG: chlorhexidine gluconate-treated rats, and talc: talc-treated rats.

+: mild, ++: moderate, and +++: severe.
